# High‐Intensity Focused Ultrasound as a Therapeutic Option to Treat Biostimulator's Nodules: A Case Report

**DOI:** 10.1111/jocd.70902

**Published:** 2026-05-05

**Authors:** Airá Novello Vilar, Vitoria Azulay, Estevão Vargas, Rubem David Azulay, Luara Lis Boson, Lismary De Forville Mesquita, Annia Cordeiro

**Affiliations:** ^1^ Institute of Dermatology Professor Rubem David Azulay Santa Casa de Misericórdia Do Rio de Janeiro Rio de Janeiro Brazil; ^2^ Private Clinics Curitiba Paraná Brazil; ^3^ Annia Cordeiro Private Practice, Dermatology Curitiba Brazil

To the Editor,

Poly‐L‐lactic acid (PLLA) is a biocompatible and biodegradable semipermanent filler with collagen‐stimulation properties. It stimulates an inflammatory response, activating fibroblasts and neocollagenesis [1]. As with any procedure, complications can occur. Although they are less frequent than those with hyaluronic acid, there is not any established protocol to treat them. Half of procedures leading to complications are performed by physicians and the most common is the presence of late nodules (89,1%) that appear after a period of 1 month. Different treatments are proposed, such as injecting saline solution, 5‐fluorouracil, intralesional corticosteroids, and, more recently, microfocused ultrasound (microfocused‐usg) [2].

We herein present a case of a 50‐year‐old female patient, without comorbidities, who underwent a procedure with PLLA Elleva with a final dilution solution of 18 mL, following the manufacturer's protocols.

After 5 months, the patient presented with biostimulatory filler‐induced palpable nodules in the neck (Figure 1a). She was submitted to a biopsy that evidenced chronic granulomatous inflammatory reactions with foreign body‐type giant cells containing exogenous material in the cytoplasm of macrophages. The PLLA's material stained positively in the Alcian blue staining—due to acidic mucins in the vehicle [1] (Figure 1b).

**FIGURE 1:**
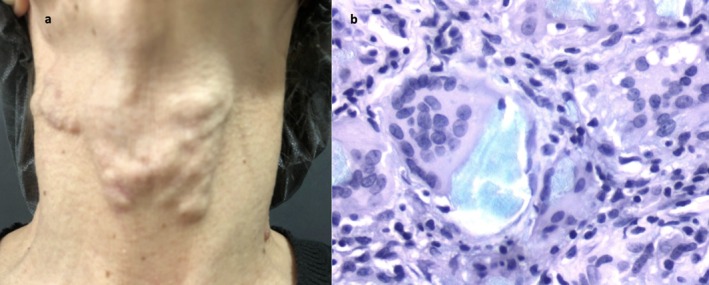
(a) Palpable nodules on the neck. (b) Histopathology (Alcian Blue staining 400×) showing chronic granulomatous inflammatory reactions with foreign body‐type giant cells. Elleva stained positively in the Alcian blue staining.

After multiple prior treatment attempts with intralesional corticosteroids and saline solution, a different therapeutic modality was attempted: microfocused‐usg.

It is important to acknowledge that these prior interventions may have partially contributed to the clinical outcome. However, the persistence of nodules before microfocused‐usg suggests an additional therapeutic effect of this modality.

The patient underwent a total of 15 treatment sessions at 20‐day intervals. Each session was performed using two transducers: a 3.0‐mm depth transducer delivering 0.5 J per line and a 1.5‐mm depth transducer delivering 0.4 J per line. The applications were targeted to the clinically palpable nodules, with approximately 300 lines delivered per session (Figure 2a). The use of two different depths aimed to target both superficial and deeper components of the nodules, optimizing energy delivery according to lesion depth in order to ensure that the center of energy delivery corresponded to the center of each lesion. Proper alignment is essential to avoid targeting the periphery of the nodule, which may lead to unintended fibroblast stimulation and potential worsening due to peripheral fibrosis.

**FIGURE 2:**
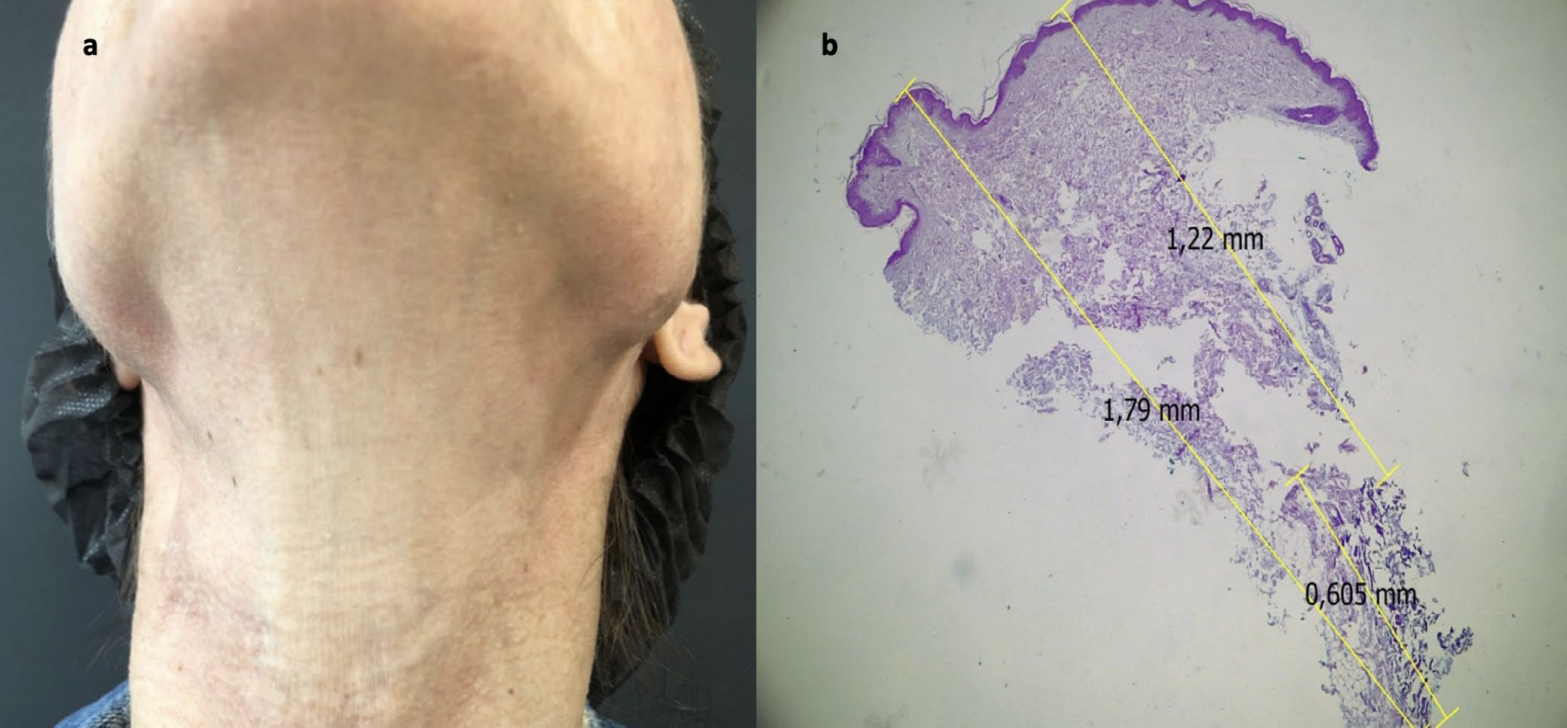
(a) After treatment. (b) Histopathology (hematoxylin and eosin staining 4×) demonstrating the distance 1.22–1.79 mm with the use of a 1.5‐mm transducer (center of the shot with repercussion in a ray in three dimensions).

After a 9‐month follow‐up period, a repeat ultrasound examination revealed no remaining product. The use of this technology to treat biostimulator‐induced nodules has been described in the literature, but has been less explored. In this context, accurate assessment of nodule depth is essential to guide appropriate treatment planning (Figure 2b). It may represent a valuable therapeutic option for chronic nodules that are refractory to other treatments [3].

Microfocused‐usg is a noninvasive technology widely used in aesthetic dermatology for the treatment of tissue laxity induced by focused acoustic energy. The technique is based on the precise delivery of high‐frequency ultrasound waves concentrated at specific subdermal points, with predefined depths of penetration (1.5 mm, 3.0 mm, and 4.5 mm), according to the transducer employed. When focused, the acoustic energy generates localized thermal elevation, with estimated temperatures between 60°C and 70°C—sufficient to induce protein denaturation and coagulative necrosis within microthermal coagulation zones (TCZs), measuring between 0.5 and 1 mm. These areas of thermal injury are surrounded by zones of preserved tissue, allowing for targeted regeneration. The subsequent biological process is characterized by controlled inflammatory infiltration, activation of dermal fibroblasts, neovascularization, and de novo type I collagen deposition. This cascade results in extracellular matrix remodeling, ultimately leading to increased skin firmness and elasticity [4, 5, 6].

In this sense, energy‐based devices act by dispersing high energy in the tissue with local heat. A thermal coagulation zone is created, leading to focal tissue necrosis within the nodules. Additionally, this localized thermal effect may contribute to the degradation of the implanted material, reducing the foreign body load and consequently attenuating the associated granulomatous inflammatory response. This mechanism may partially explain the clinical improvement observed in this case.

This process, occurring in the so‐called microthermal zones, is beneficial when aimed at fibrotic nodules or residual filler material. However, when the focal zone is misaligned—particularly when the peripheral area of the filler is targeted instead of its center—microfocused‐usg may paradoxically exacerbate the condition by stimulating excessive fibroblast activity in surrounding tissues. This can lead to increased collagen deposition and progressive peripheral fibrosis. Furthermore, it is important to avoid performing microfocused‐usg during the inflammatory phase of the nodules—that is, when erythema and edema are present—as it may exacerbate these lesions.

Reported adverse effects are generally mild and transient, including erythema, edema, and discomfort. However, incorrect depth selection or misalignment of the focal zone may lead to suboptimal outcomes or even worsening due to peripheral fibrosis, reinforcing the importance of appropriate technique and patient selection.

This report suggests that microfocused‐usg may represent a promising noninvasive therapeutic option for refractory biostimulator‐induced nodules. However, further studies are needed to confirm its efficacy and to establish standardized treatment protocols.

## Author Contributions

A.N.V. and V.A. performed the research, designed the research study, and wrote the paper. E.V. wrote the paper and analyzed the data. R.D.A. wrote the paper. L.L.B., L.D.F.M, and A.C. contributed essential tools and analyzed the data. All authors have read and approved the final version of the manuscript.

## Funding

The authors have nothing to report.

## Ethics Statement

The study was approved by the Ethical Review Board.

## Conflicts of Interest

The authors declare no conflicts of interest.

## Data Availability

All data generated or analyzed during this study are included in this article. Further enquiries can be directed to the corresponding author.
